# Agreement between Xpert and AmpFire tests for high-risk human papillomavirus among HIV-positive women in Rwanda

**DOI:** 10.4102/ajlm.v11i1.1827

**Published:** 2022-10-19

**Authors:** Anthere Murangwa, Kanan T. Desai, Julia C. Gage, Gad Murenzi, Patrick Tuyisenge, Faustin Kanyabwisha, Aimable Musafili, Gallican Kubwimana, Leon Mutesa, Kathryn Anastos, Hae-Young Kim, Philip E. Castle

**Affiliations:** 1 Rwanda Military Hospital, Kigali, Rwanda; 2 Division of Cancer Epidemiology & Genetics, National Cancer Institute, National Institutes of Health, Rockville, Maryland, United States; 3 Department of Paediatrics and Child Health, School of Medicine and Pharmacy, College of Medicine and Health Sciences, University of Rwanda, Kigali, Rwanda; 4 Research for Development (RD Rwanda), Kigali, Rwanda; 5 Centre for Human Genetics, College of Medicine and Health Sciences, University of Rwanda, Kigali, Rwanda; 6 Departments of Medicine and Epidemiology & Population Health, Albert Einstein College of Medicine and Montefiore Health Systems, Bronx, New York, United States; 7 Department of Public Health, New York Medical College, Valhalla, New York, United States; 8 Division of Cancer Prevention, National Cancer Institute, National Institutes of Health, Rockville, Maryland, United States

**Keywords:** Xpert HPV assay, AmpFire HPV genotyping assay, high-risk human papillomavirus types, women living with HIV, cervical cancer screening

## Abstract

**Background:**

High-risk human papillomavirus (hrHPV) may cause more than 99% of cervical cancers worldwide. Little is known about performance differences in tests for hrHPV.

**Objective:**

This study analysed agreement for detection of hrHPV between the established, clinically validated Xpert HPV assay and the novel isothermal amplification-based AmpFire HPV genotyping assay.

**Methods:**

This study was nested in a larger project on cervical cancer screening among approximately 5000 women living with HIV in Kigali, Rwanda. This sub-study included 298 participants who underwent initial screening for cervical cancer using the Xpert HPV assay and visual inspection with acetic acid in 2017 and tested positive by either or both. Participants were rescreened using colposcopy, and cervical samples were collected between June 2018 and June 2019. Samples were then tested for HPV using the Xpert HPV assay and AmpFire HPV genotyping assay. Agreement between results from both tests was analysed using an exact version of McNemar test and chi-square test.

**Results:**

Overall agreement and kappa value for detection of hrHPV by Xpert and AmpFire were 89% and 0.77 (95% confidence interval: 0.70–0.85). AmpFire was marginally more likely to diagnose hrHPV-positive than Xpert (*p* = 0.05), due primarily to the extra positivity for HPV16 (*p* < 0.001).

**Conclusion:**

Overall, there was good to excellent agreement between the Xpert and AmpFire when testing hrHPV types among women living with HIV. AmpFire was more likely to test extra cases of HPV16, the most carcinogenic HPV type, but the clinical meaning of detecting additional HPV16 infections remains unknown.

## Introduction

High-risk human papillomavirus (hrHPV) causes virtually all cervical cancer-related cases worldwide.^1^ The elimination of cervical cancer relies on primary prevention, involving the immunisation against human papillomavirus (HPV), and on secondary prevention based on the screening and treatment of cervical precancer lesions.^2^

Evidence to date indicates that HPV genotyping can help with risk stratification for further triage and management of hrHPV-positive women.^3,4^ Unfortunately, HPV assays commonly used in the screening programmes are of higher cost, with longer turnaround time, and offer genotyping limited to HPV16 and 18 only. Many rapid, low-cost assays designed for providing extended HPV genotyping have been developed in recent decades. However, they have variable analytical sensitivity and specificity for different HPV types with limited clinical validation data.^5,6,7^

Xpert is a World Health Organization pre-qualified test,^8,9^ which uses a single integrated cartridge, prefilled with primers and fluorescent probes, as well as two reagents meant for the extraction, amplification and detection of some HPV DNA regions, on the Xpert platform. Xpert uses 1 mL of cervical specimen, which is collected in PreservCyt, to test for 14 types of hrHPV using real-time polymerase chain reaction in six color channels contain primers and probes for the detection of specific genotypes or pooled results as follows: ‘SAC: Primary’ for the Samples Adequacy Control, ‘HPV 16; Primary’ for HPV 16, ‘HPV 18_45; Primary’ for the HPV 18/45 pooled result, ‘P3: Primary’ for the pooled result of any of HPV types 31, 33, 35, 52 or 58, ‘P4: Primary’ for the pooled result of either of HPV types 51 or 59, and ‘P5; Primary’ for the pooled result of any of HPV types 39, 56, 66 or 68. The turnaround time for the assay is approximately 1 h.^3,5,6^

The AmpFire is a promising new lower-cost assay, using isothermal amplification along with a real-time fluorescence system in four channels to individually detect 15 types of hrHPV, including HPV16, 18, 31, 33 35, 39, 45, 51, 52, 56, 58, 59 and 68, plus intermediate-risk HPV53 and 66. The AmpFire assay uses a simple processing protocol to detect HPV directly without prior DNA extraction from clinical samples, including dry samples. The assay has a small footprint and a sample-to-result time of approximately 1 h.^7,10,11^

We recently completed a cervical cancer screening study of approximately 5000 Rwandan women living with HIV. In a subset of women who underwent colposcopy, we compared the analytic performance for HPV detection by the Xpert and AmpFire systems.

## Methods

### Ethical considerations

This sub-study derived from a larger study on cervical cancer screening among Rwandan women with HIV,^12^ which was approved by the Rwanda National Ethics Committee and Albert Einstein College of Medicine Institutional Review Board (no. 878/RNEC/2016). Participation in the parent study was voluntary. Women who participated in this research were informed about the study purpose and procedures before consenting to participate. Confidentiality was consistently observed when handling data.

### Study design and sampling

The participants included 298 women living with HIV who were recruited from a larger study of cervical cancer screening, which was carried out among approximately 5000 Rwandan women living with HIV. All women were initially screened by either the Xpert HPV assay (Cepheid, Sunnyvale, California, United States) or visual inspection with acetic acid and those positive by either or both methods were referred for colposcopy. A detailed protocol related to the study population and recruitment was further developed in the parent study.^12^

The participants included women who were lost to follow-up in the parent study for a period of 6–38 months. After retrieving them, they were enrolled in the present study, for which specimens were collected between June 2018 and June 2019. These women with a delay in colposcopy attendance underwent colposcopy, during which cervical specimens were collected using a LuckMedical Cervical Brush (Luck Medical Consumables Co., Jiangsu, China). These samples were put into 20 mL of the PreservCyt medium (Hologic, Bedford, Massachusetts, United States).

### Setting

All women included in the study lived in Kigali, which is the capital and biggest city of Rwanda. The latest Population and Housing Census, dated 2012, indicated that Kigali was populated with 1 132 686 inhabitants.^13^ The study was implemented at Rwanda Military Hospital, which is a tertiary referral hospital located in Kigali. The recruitment and screening of participants for HPV as well as the data collection were carried out in the department of HIV, where women living with HIV were usually followed up.

### Laboratory testing

Cervical specimens were mixed with PreservCyt before being tested for hrHPV. A volume of 1 mL of the mixed sample was added to the Xpert cartridge, which was then placed in the Xpert assay for the analysis. Results from this analysis were available within an hour.

The sample of cervical specimens mixed with PreservCyt was also tested for hrHPV using the AmpFire (Atila Biosystems, Mountain View, California, United States). A volume of 1 mL of this sample was added to a 1.5 mL-micro-centrifuge tube and centrifuged for 10 min at 10 000 revolutions per minute.^14^ The supernatant was removed from the micro-centrifuge tube while the pellet underwent further treatment by mixing it with the lysis buffer before it was incubated for 10 min at 95 °C. After the incubation, 2 µL of the treated pellet were mixed with a prepared solution of 12 µL of reaction mix and 11 µL of primer mixes (Atila Biosystems, Mountain View, California, United States), which were the AmpFire genotyping reagents. The mixed sample was placed in the AmpFire for the analysis, which took an hour before the results were available.

The cut-off point values of positivity applied when using Xpert and AmpFire assays were defined according to the manufacturers’ instructions.^15,16^

### Statistical analysis

Agreement statistics (positive, negative and overall agreement, and kappa values) for 14 HPV types, 16, 18, 31, 33, 35, 39, 45, 51, 52, 56, 58, 59, 66 and 68, detected by both tests, were calculated. Results for HPV53, detected by AmpFire but not by Xpert, were excluded. Results from AmpFire were grouped according to the HPV groups as detected by the Xpert HPV assay. Firstly, HPV groups were considered non-hierarchically, recognising that a given specimen could test positive for more than one group. Secondly, the two tests were also compared using risk-based hierarchical HPV group types, considering that different HPV groups are associated with different risks of cervical cancer: HPV16 positive, else positive for HPV18/45, else positive for HPV 31/33/35/52/58, else positive for other high-risk HPV types (HPV51/59/39/56/66/68), or else negative; HPV51/59 and HPV39/56/66/68 have similarly lower risk for invasive cervical cancer and were grouped together. McNemar test and chi-square test was applied to assess significant differences (*p* < 0.05) in test positivity. Data entry was performed using Epi-Info 7 (version March 2015, Centers for Disease Control, Atlanta, Georgia, United States). Afterwards, data were transferred to the Statistical Package for the Social Sciences Statistics 20 (IBM Corp., Armonk, New York, United States) for the analysis.

## Results

Overall agreement between the two test results for the 14 HPV types was 89% and the kappa value was 0.77 (95% confidence interval: 0.70–0.85). AmpFire was marginally more likely to test positive for the 13 types of HPV (*p* = 0.05) than Xpert (Table 1). Agreement ranged from 92% (HPV31/33/35/52/58) to 98% (HPV51/59), and kappa values ranged from 0.68 (HPV39/56/66/68) to 0.84 (HPV18/45) for HPV groups. AmpFire was more likely to test positive for HPV16 than Xpert (*p* < 0.001).

**TABLE 1:** Agreement for detection of human papillomavirus overall and HPV types grouped according to the Xpert HPV assay between Xpert HPV assay and AmpFire HPV genotyping assay, Rwanda, June 2018 - June 2019.

HPV results AmpFire/Xpert	+/+		−/+		+/−		−/−		Positive agreement (%)	Negative agreement (%)	Overall agreement (%)	Exact *p*-value for asymmetry	Unweighted kappa	95% confidence interval
	*n*	%	*n*	%	*n*	%	*n*	%						
HPV16	35	12.4	1	0.4	18	6.4	229	80.9	65	92	93	< 0.001	0.75	0.64–0.85
HPV18/45	27	9.5	3	1.1	6	2.1	247	87.3	75	96	97	0.510	0.84	0.74–0.94
HPV31/33/35/52/58	66	23.3	10	3.5	12	4.2	195	68.9	75	90	92	0.830	0.80	0.72–0.88
HPV51/59	13	4.6	1	0.4	6	2.1	263	92.9	65	97	98	0.130	0.77	0.61–0.94
HPV39/56/66/68	23	8.1	6	2.1	12	4.2	242	85.5	56	93	94	0.240	0.68	0.55–0.82
HR13† + HPV 66	134	47.3	10	3.5	22	7.8	117	41.3	81	79	89	0.050	0.77	0.70–0.85

Note: *N* = 283 women living with HIV undergoing colposcopy (non-hierarchical).

HPV, human papillomavirus.

† , HR13 includes HPV16, 18, 31, 33, 35, 39, 45, 51, 52, 56, 58, 59 and 68.

On the hierarchical analysis, the two assays had good agreement with an overall unweighted kappa value of 0.77 (95% confidence interval: 0.70–0.85) (Table 2). The hierarchical analysis also showed a similar pattern to that of non-hierarchical analysis for HPV 16 and HPV 18/45, with extra cases detected by AmpFire. However, for HPV31/33/35/52/58, more extra cases were detected by Xpert than by AmpFire while for other hrHPV types, both tests detected similar numbers of extra cases. Overall, AmpFire was more likely to rank HPV results in categories with higher risk than Xpert (*p* < 0.001).

**TABLE 2:** Pairwise AmpFire and Xpert results, categorised hierarchically according to HPV risk groups, Rwanda, June 2018 - June 2019.

Xpert AmpFire	HPV16	HPV18/45	HPV31/33/35/52/58	HPV51/59 + HPV39/56/66/68	Negative	Total
HPV16	**35**	2	5	4	7	53
Row %	**66.0**	3.8	9.4	7.5	13.2	100.0
Column %	**97.2**	7.1	8.5	19.0	5.0	18.7
HPV18/45	0	**24**	3	1	2	30
Row %	0.0	**80.0**	10.0	3.3	6.7	100.0
Column %	0.0	**85.7**	5.1	4.8	1.4	10.6
HPV31/33/35/52/58	0	0	**44**	0	8	52
Row %	0.0	0.0	**84.6**	0.0	15.4	100.0
Column %	0.0	0.0	**74.6**	0.0	5.8	18.4
HPV51/59 + HPV39/56/66/68	0	0	0	**16**	5	21
Row %	0.0	0.0	0.0	**76.2**	23.8	100.0
Column %	0.0	0.0	0.0	**76.2**	3.6	7.4
Negative	1	2	7	0	**117**	127
Row %	0.8	1.6	5.5	0.0	**92.1**	100.0
Column %	2.8	7.1	11.9	0.0	**84.2**	44.9
Total	36	28	59	21	139	**283**
Row %	12.7	9.9	20.8	7.4	49.1	**100.0**
Column %	100.0	100.0	100.0	100.0	100.0	**100.0**

Note: *N* = 283 women living with HIV undergoing colposcopy. Data in bold are concordant HPV risk group results.

Unweighted kappa = 0.77 (95% confidence interval = 0.70–0.85).

HPV, human papillomavirus.

More than 80% of the HPV18/33/35/45/52/58/59 detected by AmpFire were positive in the Xpert channel that tested for that type (as a group of types) (Table 3). By contrast, only 54.4% of HPV51 and 42.9% of HPV39 detected by AmpFire were positive in the Xpert channel that tested for that type (as a group of types).

**TABLE 3:** Concordance of individual HPV types detected by AmpFire HPV genotyping assay with HPV groups detected by Xpert HPV assay (non-hierarchical), Rwanda, June 2018 - June 2019.

AmpFire positive	AmpFire positive confirmed on the Xpert channel for respective types	
	*n*	%
HPV16 (*n* = 53)	35	66.0
HPV18 (*n* = 19)	16	84.2
HPV45 (*n* = 16)	13	81.3
HPV31 (*n* = 14)	11	78.6
HPV33 (*n* = 22)	19	86.4
HPV35 (*n* = 16)	16	100.0
HPV52 (*n* = 23)	20	87.0
HPV58 (*n* = 19)	16	84.2
HPV51 (*n* = 11)	6	54.4
HPV59 (*n* = 8)	7	87.5
HPV39 (*n* = 7)	3	42.9
HPV56 (*n* = 14)	10	71.4
HPV66 (*n* = 8)	6	75.0
HPV68 (*n* = 8)	6	75.0
Negative (*n* = 127)	117	92.1

Note: Combining the AmpFire results to match the Xpert HPV groups, in 283 women living with HIV undergoing colposcopy.

HPV, human papillomavirus.

## Discussion

This study sought to compare the Xpert and AmpFire among women living with HIV in Rwanda. We found an overall good to excellent agreement between the two methods for the detection of hrHPV. Notably, AmpFire was more likely to test positive cases for HPV16 than Xpert but the clinical meaning of the detection of these additional HPV16 infections was unknown.

The comparable findings between Xpert and AmpFire HPV regarding the detection of hrHPV may be explained by the well-known performance of these tests. Previous studies have shown that AmpFire could detect all HPV genotypes with sensitivity and specificity of more than 95%.^5^ In addition, when compared to other tests, including the Roche cobas^®^ and Linear Array, an excellent concordance between these tests and AmpFire has been demonstrated for the detection of 15 hrHPV.^16^

Further, Xpert has been widely used for the detection of various types of HPV, including hrHPV types. In Zimbabwe, an overall good concordance (77.2%, kappa = 0.698) between Xpert and Seegene Anyplex II HPV HR detection kit was found when testing hrHPV among women aged from 30 to 60 years.^17^ In South Africa, Xpert was combined with Linear Array to optimise the screening of cervical cancer. This combination has resulted in increased sensitivity and specificity for the detection of hrHPV, highlighting the consistent high performance of Xpert even when combined with other valid tests.^15^ The Xpert was also considered a World Health Organization pre-qualified test, stressing its validity.^9^

Despite the overall agreement between the Xpert and AmpFire, differences in the detection of some types of hrHPV were noticed. These differences could be reflected by the extra cases of HPV16 and other hrHPV, including HPV18 and 45, which were detected by the AmpFire compared to Xpert. However, we could not find any scientific reason that could be attributed to the extra positivity by AmpFire, especially for HPV16. In contrast, Xpert has shown greater potential of detecting more cases of HPV31/33/35/52/58 than AmpFire. This extra detection of some hrHPV by Xpert compared to AmpFire could probably be explained by its greater performance of detecting concurrent infections by hrHPV.

### Recommendations

As explained earlier, both assays perform as expected and use simple procedures with less equipment, and a rapid turnaround time, which make them highly recommendable as point-of-care screening for cervical precancer and cancer in low- to middle-income countries.

### Strengths

This was the first study to have analysed the agreement between Xpert and AmpFire for the detection of hrHPV among women living with HIV in Rwanda. Thus, our findings may provide new insights regarding the performance of the two assays when applied in low-resource settings.

### Limitations

The authors could not determine why there was extra positivity by AmpFire, especially for the detection of HPV16. Further studies, involving larger numbers of specimens and multiple sites, or histopathologic analyses could perhaps be relevant to address this knowledge gap.

### Conclusion

This study revealed good to excellent agreement between Xpert and AmpFire when testing hrHPV types among women living with HIV in a Rwandan setting. It has also shown an extra positivity by AmpFire compared to Xpert, especially for HPV16, which requires further studies or adjudication by histopathologic tests.
